# Directed Expression of Tracheal Antimicrobial Peptide as a Treatment for Bovine-Associated *Staphylococcus Aureus*-Induced Mastitis in Mice

**DOI:** 10.3389/fvets.2021.700930

**Published:** 2021-10-04

**Authors:** Zhipeng Zhang, Daijie Chen, Xubin Lu, Ruifeng Zhao, Zhi Chen, Mingxun Li, Tianle Xu, Yongjiang Mao, Yi Yang, Zhangping Yang

**Affiliations:** ^1^College of Animal Science and Technology, Yangzhou University, Yangzhou, China; ^2^Joint International Research Laboratory of Agriculture & Agri-Product Safety, Ministry of Education, Yangzhou University, Yangzhou, China; ^3^Jiangsu Co-innovation Center for Prevention and Control of Important Animal Infectious Diseases and Zoonoses, College of Veterinary Medicine, Yangzhou University, Yangzhou, China

**Keywords:** bovine mastitis, *TAP*, antibiotic alternatives, *S. aureus*, mice model, plasmid

## Abstract

Bovine mastitis is perplexing the dairy industry since the initiation of intensive dairy farming, which has caused a reduction in the productivity of cows and an escalation in costs. The use of antibiotics causes a series of problems, especially the formation of bacterial antimicrobial resistance. However, there are limited antibiotic-free therapeutic strategies that can effectively relieve bacterial infection of bovine mammary glands. Hence, in this study, we constructed a mammary gland tissue-specific expression vector carrying the antimicrobial peptide of bovine-derived tracheal antimicrobial peptide (*TAP*) and evaluated it in both primary bovine mammary epithelial cells (pBMECs) and mice. The results showed that the vector driven by the β*-*lactoglobulin gene (*BLG*) promoter could efficiently direct the expression of *TAP* in pBMECs and the mammary gland tissue of mice. In addition, significant antibacterial effects were observed in both *in vitro* and *in vivo* experiments when introducing this vector to bovine-associated *Staphylococcus aureus*-treated pBMECs and mice, respectively. This study demonstrated that the mammary gland tissue-specific expression vector could be used to introduce antimicrobial peptide both in *in vitro* and *in vivo* and will provide a new therapeutic strategy in the treatment of bovine mastitis.

## Introduction

Cow mastitis is one of the most prevalent domestic animal diseases and leads to reduced milk production, increased veterinary cost, and early culling (1–3). The primary reason for mastitis is bacterial infection; one of the most problematic pathogens is *Staphylococcus aureus*, a Gram-positive pathogen that infects between 3 and 15% of dairy cows in a herd (4). At present, to reduce economic losses, most farms set up mastitis management programs, such as routine antibiotic therapy, culling cows with chronic infections, and monitoring milk somatic cell numbers (5). For breeding mastitis-resistant dairy cattle, approaches through both traditional breeding methods, including comparing innate immune response patterns and metabolic parameters, and recent studies to find molecular markers through epigenetic and genome sequencing are attempting to improve cow resistance to mastitis (6). However, these approaches still have various limitations. For example, the routine use of antibiotics may lead to antibiotic resistance (7). Cow breeds from the mastitis resistance breeding procedure can hardly meet all economic parameters perfectly, such as a decrease in milk production (6). Mastitis is a lasting and difficult problem that still challenges the global dairy industry.

Antibiotic-free approaches aimed at curing or relieving mastitis symptoms have been investigated to overcome these obstacles (8). Studies on therapeutic strategies with bacteriophages (9), nanoparticles (10), cytokines (11), natural compounds (12), and antimicrobials (13) have the potential to become novel effective methods. β-defensins are potential antimicrobial that can protect against respiratory pathogens in cattle (14). It is mainly distributed in the mucosal layer of various organs of mammals and birds, and is also widely expressed in breast tissue, which plays an important role in breast resistance to microbial infection and maintenance of normal physiological functions (15). The biological activity, expression regulation, and genetic engineering of defensins have always been the focus of research. Tracheal antimicrobial peptide, a β-defensin, was firstly discovered in 1991 (16). It has a broad spectrum of antimicrobial activity and special antimicrobial mechanism. Both pathogenic microorganisms and various pro-inflammatory factors can specifically induce the upregulation of *TAP* gene expression in mammary tissue of dairy cows, with the maximum upregulation of tens of times, thus enhancing the local defense ability of mammary gland (17). Therefore, tracheal antimicrobial peptide is expected to provide a powerful help to solve the growing problem of bacterial resistance.

Here, we developed a plasmid transgene strategy that results in specific expression of *TAP* in bovine mammary gland cells. Plasmid-delivered *TAP* significantly relieved *S. aureus* infection, and the plasmid therapy effect was verified in mice. This research established a new potential antimicrobial therapy for bacterially derived bovine mastitis.

## Materials and Methods

### Ethics Statement

All experimental protocols in this study were reviewed and approved by the Institutional Animal Care and Use Committee of Yangzhou University (ZZCX2020-SYXY-2). All methods in this study were carried out in accordance with the Administration of Affairs Concerning Experimental Animals published by the Ministry of Science and Technology of China.

### Vector Construction

**Figure 1:**
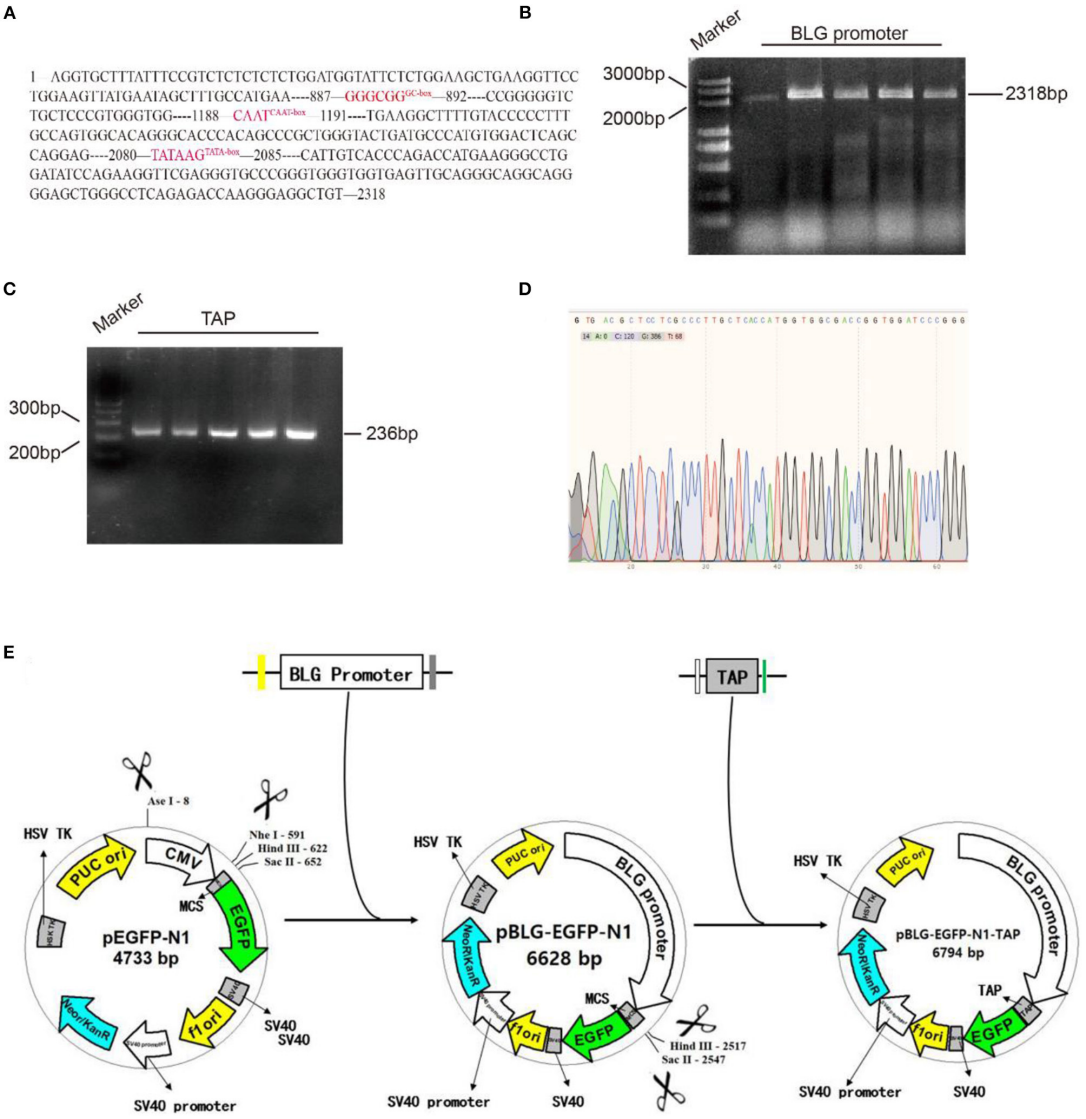
Molecular cloning of pBLG-TAP-EGFP-N1. **(A)** Analysis diagram of the promoter element of *BLG*. **(B)** *BLG* promoter PCR amplification diagram (2,358 bp corresponds to the 2,318 bp promoter + 20 bp upstream homologous arm + 20 bp downstream homologous arm). Marker: DL5000 DNA Marker. BLG promoter: PCR amplification product of *BLG* promoter. The five rows contained the same sample with replicates. **(C)** PCR amplification of the *TAP* gene (236 bp corresponds to the 192 bp *TAP* gene + 22 bp upstream homologous arm + 22 bp downstream homologous arm). Marker: GeneRuler™ 100 bp DNA Ladder. TAP: PCR amplification product of *TAP* gene. The five rows contained the same sample with replicates. **(D)** The recombination vector named pBLG-TAP-EGFP-N1 was verified by sequencing. The result showed that all of the target genes (*TAP* gene, *BLG* promoter) were correct without any misalignment, indicating that pBLG-TAP-EGFP-N1 was successfully constructed. **(E)** Design and construction of pBLG-TAP-EGFP-N1.

Plasmid pEGFP-N1 (Miaoling Biosystem, Wuhan, China) was used as the basal plasmid in the construction of mammary gland tissue-specific expression vector carrying *TAP* gene. The bovine-derived *TAP* and *BLG* promoter genes were cloned from the cDNA and DNA extracted from the whole blood and pBMECs of a Holstein cow, respectively. pBLG-EGFP-N1 was generated by replacing the *Ase*I–*Nhe*I fragment (cytomegalovirus promoter) with the *BLG* promoter using homologous recombination (Thermo Fisher, Shanghai, China). In addition, a *Hind*III–*Sac*II fragment that contained *TAP* sequence was ligated into the multiple cloning site (MCS) of pBLG-EGFP-N1 to obtain the plasmid pBLG-TAP-EGFP-N1 (Figure 1E). Primers used in the construction of pBLG-TAP-EGFP-N1 are shown in Table 1.

**Table 1:** Introduction of primer information.

**Gene**	**Size**	**Primer sequence (5^**′**^-3^**′**^)**
*TAP*	236 bp	F:CTCAGATCTCGAGCTCAAGCTT ATGAGGCTCCATCACCTGCT
		P:CATGGTGGCGACCGGTGGATCC CTTCTTTCTACAGCATTTTA
*BLG*promoter	2,518 bp	F:GCCATGCATTAGTTATTAAT AGGTGCTTTATTTCCGTCTC
		P:GACTGCAGAATTCGAAGCTT ACAGCCTCCCTTGGTCTC
*Nuc*	279 bp	F:GCGATTGATGGTGATACGGTT
		R:AGCCAAGCCTTGACGAACTAAAGC

*The underlined part is the homologous sequence of the target knockout gene*.

### Cells and *in vitro* Transfection

Primary bovine mammary epithelial cells (pBMECs) were aseptically obtained from a healthy slaughtered Holstein lactating cow and maintained in DMEM/F12 medium (Thermo Fisher, Waltham, USA) supplemented with 10% heat-inactivated fetal bovine serum (Thermo Fisher, Beijing, China), penicillin–streptomycin (Procell, Wuhan, China), and various cytokines as previously described (18). For transient transfection, 1 μg of either pBLG-TAP-EGFP-N1 vector or negative controls (Novus Biologicals, Briarwood, USA) was used to transfect pBMECs and HEK293T cells with FuGENE^®^ 6 Transfection Reagent (Promega, Madison, USA). At 12 h after transfection, the expression of *GFP* was observed and photographed under an inverted fluorescence microscope (Carl Zeiss, Shanghai, China).

### The Isolation of Bovine-Derived *S. aureus*

The strain of *S. aureus* used in this study was isolated from raw milk of a Holstein cow suffering from clinical mastitis. In short, 100 μl of diluted milk samples was plated onto a Baird-Parker Agar plate and incubated aerobically at 37°C for 12–24 h. The black and round colonies with a halo were picked for further isolation and identification. The suspected colonies were sub-cultured by streaking on a MacConkey plate, and one more optional sub-culture was conducted if different morphological colonies grew on a plate. Single colonies were identified by specific PCR targeting *Nuc* gene of *S. aureus* (Table 1) and sequencing. Subsequently, the identified colonies were expanded cultured routinely at 37°C in brain–heart infusion (HARDY Diagnostics, Santa Maria, USA) broth and stored in 30% glycerol at −80°C.

*S. aureus* was resuscitated in LB culture medium and cultured for 16 h at 37°C. The culture was centrifuged for 15 min at 5000 r/min. *S. aureus* was resuspended in a saline solution. The number of bacteria was subsequently measured in the inoculum through measurement of OD_600nm_ on a spectrophotometer (19). Based on these OD results, the inoculum was diluted in PBS to the desired number of bacteria. The exact number of CFU of the inoculum was confirmed by plate counts on tryptone soya agar plates.

### Bacterial Challenge

The pBMECs were seeded in six-well plates at 80% confluence before infection, and the cells were divided into three groups: a normal group, an infection group, and an antibacterial group. The cells in the infected group were incubated with bovine-associated *S. aureus* for 4 h at 37°C and with 5% CO_2_ and a multiplicity of infection (MOI) of 100:1 from the infected group. The cells in the antibacterial group were infected with bovine-associated *S. aureus* 12 h after transfection with the recombinant plasmid. All groups of cells were collected from six-well plates and fixed overnight in chilled 70% ethanol (−20°C). The cells were centrifuged and washed two or three times with PBS. Then, 500 μl of PI staining solution (50 μg/ml propidium iodide, 100 μg/ml RNase A, and 0.2% Triton X-100) was added, and the cells were incubated at 4°C for 30 min. Finally, the cell proliferation and apoptosis were determined with CCK-8 assay (Abcom, Shanghai, China) and flow cytometry (Abcom, Shanghai, China).

### Animals

The ICR mice employed in this study were all at first parity and 7–10 days of lactation with a weight of 40–50 g. The baby mice were separated from their mothers 1–2 h before the experiment. A mixture of oxygen and isoflurane (2–3%) was used for inhalational anesthesia of the mice and a bolus of PBS-diluted Vetergesic (i.e., buprenorphine 10 μg/kg, Val d'Hony Verdifarm NV, Belgium) was administered intraperitoneally (i.p.) as analgesic prior to any surgical intervention. Mice were housed individually in a regulated environment of humidity and temperature (12-h light/dark cycle, lights on at 08:00) with standard mouse diet and water.

### *In vivo* Transfection/Infection of Mice

A total of 18 ICR mice were randomized into six groups. Three mice were first transfected with pBLG-TAP-EGFP-N1 and then challenged with *S. aureus* after 24 h, serving as the prevention group. Three mice were first challenged with *S. aureus* and then transfected with pBLG-TAP-EGFP-N1 after 24 h, serving as the treatment group. Three mice only infected with *S. aureus* served as the infection group. Three mice were inoculated with PBS and served as control group 1. Three mice were transfected with empty vector (pEGFP-N1), serving as control group 2. The remaining three mice were first challenged with *S. aureus* and then transfected with pEGFP-N1 after 24 h, serving as control group 3. For the transfection, the recombinant plasmid pBLG-TAP-EGFP-N1 was transiently transfected into mice using in Entranster-*in vivo* transfection reagent (Engreen Biosystem, Beijing, China) according to the manufacturer's instructions. Numerous studies have shown that the transfection agent causes limited inflammation in mice and rats (20, 21). In brief, the mice in the prevention and treatment groups were injected with 150 μl of transfection master mix containing 18.75 μg of DNA, 37.5 μl of transfection reagent, 75 μl of 10% glucose solution, and 37.5 μl of double-distilled water, *via* caudal vein. For the challenge of *S. aureus*, 50 μl of bacterial suspensions (1 × 10^8^ CFU/ml) were injected into the mice in the prevention, treatment, and infection groups through intramammary infusion (22). All mice were euthanized by cervical dislocation after treatments, and the mammary gland, heart, liver, spleen, lung, and kidney samples were aseptically removed for fluorescence quantitative PCR and Western blot. Breast tissue samples were also used to make paraffin sections for pathological tests.

### Quantitative Real-Time Polymerase Chain Reaction (PCR)

Total RNA was extracted from mammary gland, pBMECs, and HEK293T cells with RNAprep Pure Plant Kit (TIANGEN, Shanghai, China). The concentration and purity of the extracted RNA were detected by a spectrophotometer (Takara, Shanghai, China) to ensure the quality of RNA. The primers designed for quantitative real-time PCR (qRT-PCR) targeting *TAP* and β*-actin* (housekeeping gene) were synthesized by a commercial company (Takara, Dalian, China). qRT-PCR was performed using the LightCycler^®^ 480 System (Roche, Basel, Switzerland) with High Capacity cDNA Reverse Transcription Kits (Applied Biosystems, Foster City, USA) and SYBR Premix EX Taq (Takara, Beijing, China) according to the manufacturer's instructions. The relative expression levels of *TAP* were calculated with the 2^−ΔΔCt^ method (23).

### Western Blot

Euthanized mice had their mammary gland tissue removed, and 30 mg of mammary gland tissue was digested with 300 μl of RIPA buffer (Thermo Fisher, Shanghai, China) under ultrasound exposure for 30 min at 4°C. The samples were centrifuged at 13,800 × *g* for 20 min at 4°C. After isolating proteins by PAGE, the membranes were blocked with 5% pure milk for 2 h at room temperature (RT) and then washed with TBST (Thermo Fisher, Shanghai, China). The membranes were incubated with anti-EGFP (Biorbyt, Wuhan, China) and anti-β-actin primary antibodies (Cell Signaling Technology, Shanghai, China) overnight at 4°C. Then, the membranes were incubated with a secondary antibody for 1 h. ECL (Thermo Fisher, Shanghai, China) was used for substrate coloration.

### Bacteria Counting

Bacterial colony-forming unit (CFU) counts were obtained after plating serial logarithmic dilutions of mammary gland homogenates. The mammary gland homogenate supernatant from each sample was inoculated in a *S. aureus* chromogenic culture (Ao Bo Xing, Beijing, China) dish for bacterial culture, and the number of colonies in the culture dish was counted after 24 h, and transforming counts into base 10 logarithm (log_10_) values (24). Bacterial genomic DNA was extracted and purified from the mammary gland homogenate, and specific PCR amplification and sequencing were performed for the specific *Nuc* gene of *S. aureus* to verify that the infecting bacteria were indeed bovine-associated *S. aureus*.

### Pathological Tests

Mammary gland samples were fixed in 4% paraformaldehyde, covered with paraffin, and then dehydrated in a concentration gradient of ethanol. HE staining was performed after sectioning, and then pathological damage to the breast tissue was evaluated by optical microscopy.

### Inflammatory Cytokine Assay

The levels of TNF, IL1, and IL6 in the mammary tissues of mice were determined using enzyme-linked immunosorbent assay (ELISA) kits (Qiao Du, Shanghai, China), and the operation was performed according to the manufacturer's instructions. In brief, breast tissues were ground up after adding normal saline at 0.2 g/mL. The supernatant was centrifuged at 3,000 rpm for 10 min to detect inflammatory factors.

### Statistics and Data Analysis

All the variance of the experimental data was analyzed using SPSS 17.0 statistical software (IBM Corporation, Armonk, NY, USA). If the result was significant, the LSD method was used for multiple comparisons, and each group of data is represented by the mean ± standard deviation.

## Results

### Validation of Recombinant Plasmid pBLG-TAP-EGFP-N1

To specifically express the *TAP* gene in bovine mammary glands, we selected the promoter of *BLG* gene, which is specifically expressed in the mammary gland. The sequence of promoter region (2318 bp) was obtained from online public database GenBank (Figure 1A). It included the first 1,2 introns and exon regions of nearly 1,000 bp of *BLG* gene, as well as 2,171 bp upstream regulatory sequence at the 5' end, which contained multiple regulatory action sites or response elements, such as GC-box, CAAT-box, and TATA-box. The *BLG* promoter and *TAP* gene were cloned by PCR, and their sizes were identified through agarose gel electrophoresis. The electrophoresis results showed that the cDNA sequence of *TAP* was consistent with the expected fragment size at about 200 bp, and the *BLG* promoter sequence was consistent with the expected fragment size at about 2,000 bp (Figures 1B,C). The recombinant vector was named pBLG-TAP-EGFP-N1 and verified by Sanger DNA sequencing. After assembly and alignment, it was shown that the open reading frames (ORFs) of the recombinant plasmid showed that all target genes were correct without any misalignment, indicating that pBLG-TAP-EGFP-N1 was successfully constructed (Figure 1D).

### The Expression of pBLG-TAP-EGFP-N1 in pBMECs

**Figure 2:**
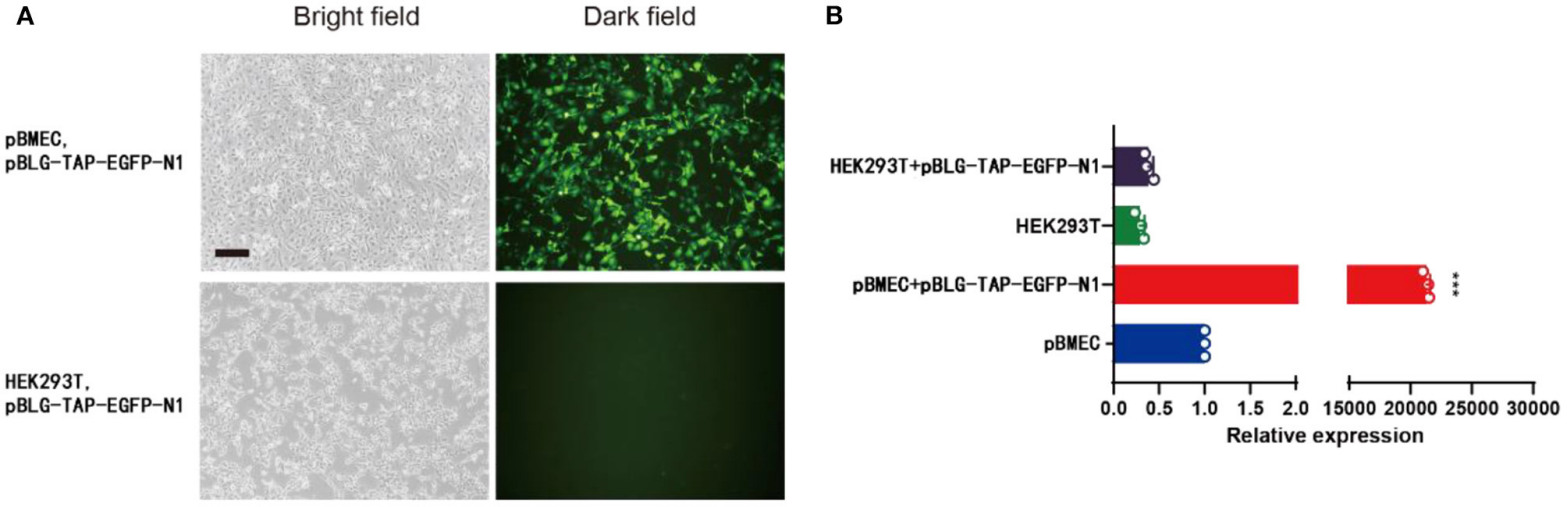
pBLG-TAP-EGFP-N1 in pBMECs. **(A)** Specific expression of pBLG-TAP-EGFP-N1 in primary mammary epithelial cells (×100 magnification). **(B)** Relative expression of the *TAP* gene in pBMECs and HEK293T cells.

The pBMECs were isolated from the collected mammary tissue of lactation cows by digestion method. The cells were pebbly and polygonal. It was observed that EGFP was efficiently expressed in pBMECs, while its expression was hardly to be detected in HEK293T cells *via* immunofluorescence after lipofection (Figure 2A). In addition, qPCR showed that a highly significant higher relative expression level of *TAP* was detected in pBMECs transfected with pBLG-TAP-EGFP-N1 (24,202.00 ± 978.73), compared with the controls (Figure 2B).

### Isolation and Identification of *S. aureus*

**Figure 3:**
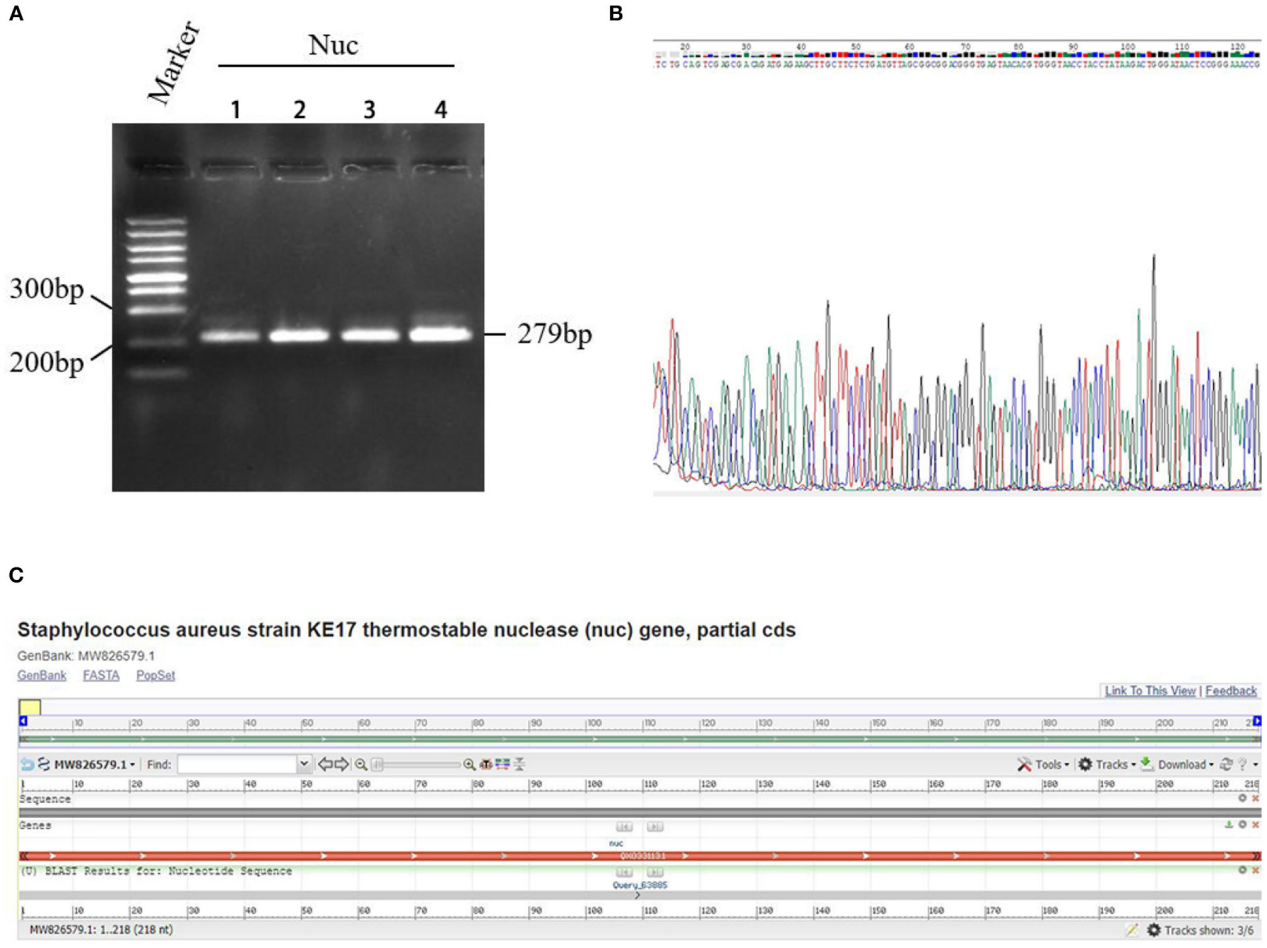
Detection results of a specific gene *Nuc* in *Staphylococcus aureus*. **(A)** PCR electrophoresis of *Nuc* gene. Marker: GeneRuler™ 100 bp DNA Ladder. Nuc: PCR amplification product of Nuc gene. **(B)** Sequencing of the Nuc gene. The four rows indicated samples of *S. aureus* isolates numbered 1–4, respectively. **(C)** The results of BLAST homology comparison showed 100% similarity with the specific *Nuc* gene of *S. aureus* in the database.

After culture and sub-culture, regular PCR targeting *Nuc* gene was performed on four suspected strains of *S. aureus*. The amplification of the 279-bp sequences of *Nuc* gene was observed from the PCR products of all the four suspected strains (Figure 3A). In addition, the sequencing data showed that these four bacterial strains belonged to *S. aureus* (Figures 3A,B) with 100% identity, and one of them (No. 4) was employed in further experiments. The results of BLAST homology comparison showed 100% similarity with the specific *Nuc* gene of *S. aureus* in the database (Figure 3C).

### The Expression of TAP Relieved *S. aureus* Infection *in vitro*

**Figure 4:**
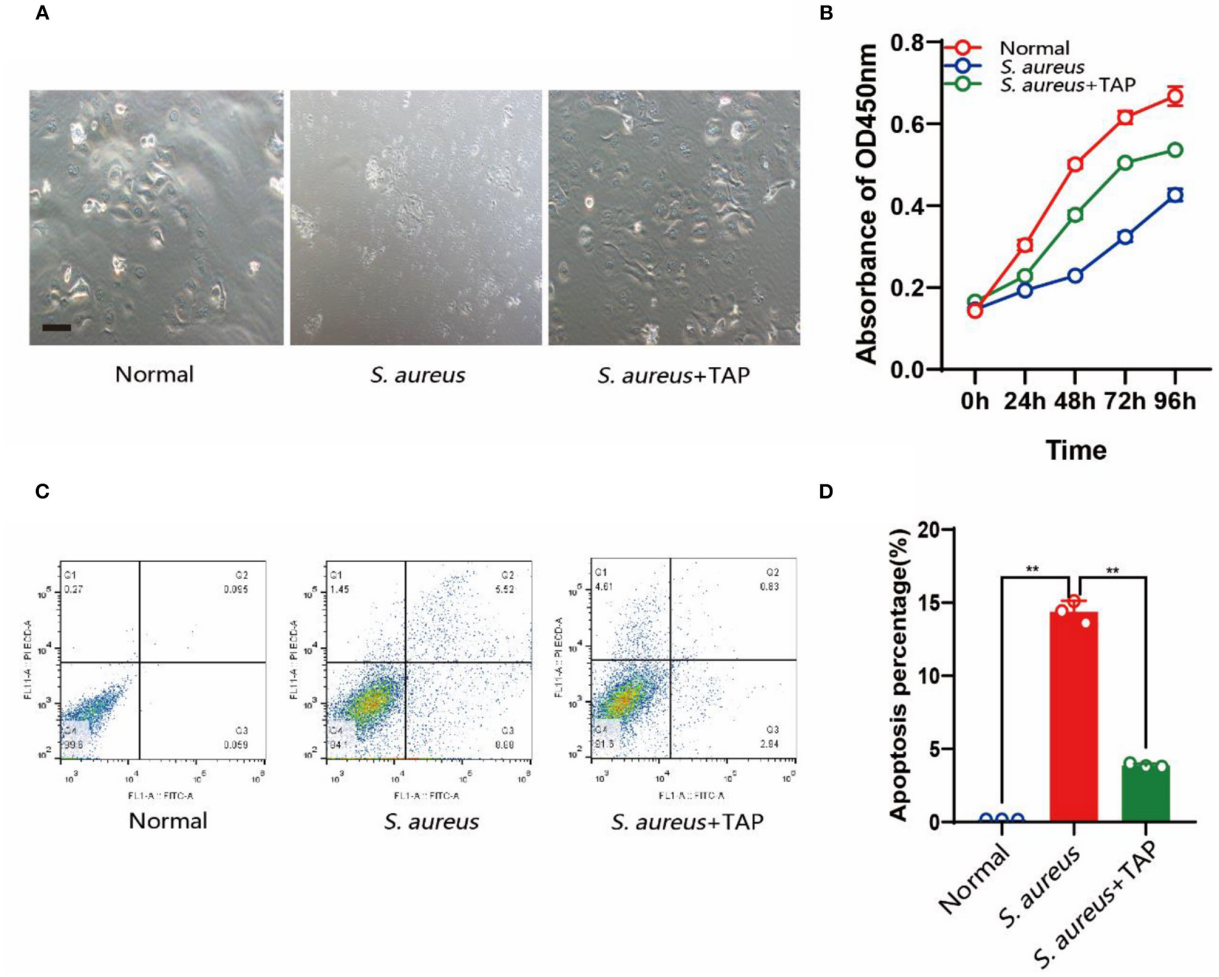
Detection of cell growth and apoptosis after pBLG-TAP-EGFP-N1 transfection. **(A)** Cell morphology after *S. aureus* and *TAP* treatment. Scale bar: 200 μm. **(B)** CCK-8 test of cell growth after treatment. **(C,D)** Cell apoptosis analysis by flow cytometry.

Under *S. aureus* challenge, pBMECs showed apoptosis characteristics, such as shrinking of the cell membrane and loss of normal cell morphology. However, with the expression of *TAP*, little change was observed in the transfected cells compared with the controls (Figure 4A). The CCK-8 assay indicated that the overexpression of *TAP* significantly relieved the decreased cell proliferation caused by *S. aureus* infection (Figure 4B). Additionally, the results of Annexin V-FITC-based flow cytometry elucidated the results above, in which the expression of TAP reduced apoptosis of infected pBMECs (Figures 4C,D). Compared with the infection group, the proportion of early apoptotic cells in the antibacterial group decreased from 8.88 to 2.94%, and the proportion of late apoptotic cells decreased from 5.52 to 0.83%. Although the rate of cell necrosis increased from 1.45 to 4.61%, the total living cell rate increased from 84.1 to 91.6%.

### The Expression of pBLG-TAP-EGFP-N1 in Mammary Tissue of Mice

**Figure 5:**
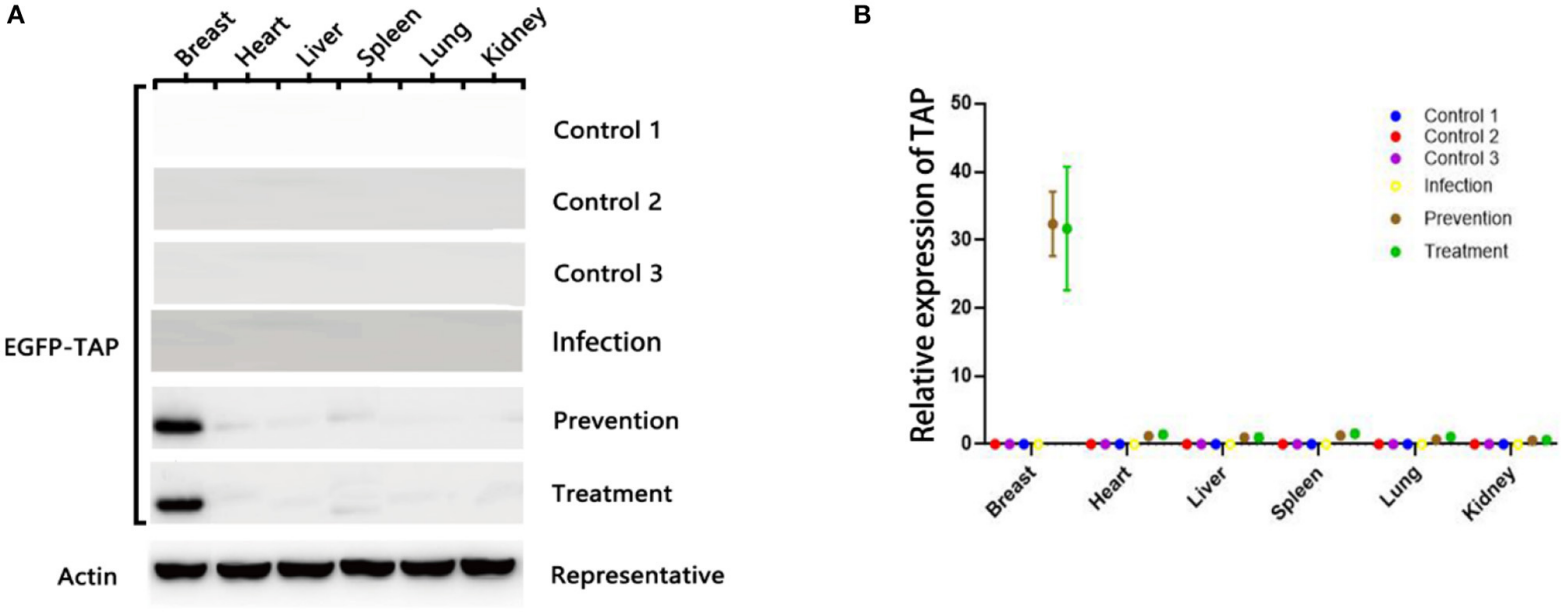
pBLG-TAP-EGFP-N1 in the mammary gland tissue of mice. **(A)** Western blot analysis of TAP expression in the mouse any tissue. The fusion protein EGFP-TAP was efficiently expressed in the mammary tissues of the treatment group (*n* = 3), while it was unable to be detected in other tissues of the transfected mice and the mammary tissues of the controls (*n* = 3). **(B)** qPCR test of *TAP* expression in mouse any tissue. The relative expression level of *TAP* gene in murine mammary tissues was significantly increased. The expression levels of target gene in the heart, liver, spleen, lung, and kidney of mice were not significantly different from that of the controls.

The plasmid pBLG-TAP-EGFP-N1 was delivered through murine caudal vein before/after *S. aureus* infection. The fusion protein EGFP-TAP was efficiently expressed in the mammary tissues of the mice transfected with the recombinant plasmid, while it was unable to be detected in other tissues of the transfected mice and the mammary tissues of the controls (Figure 5A). The real-time PCR also showed that the target gene was only transcribed in mammary tissues of transfected mice (Figure 5B). The relative expression level of *TAP* gene in murine mammary tissues was significantly increased by 32.34 ± 1.21 times (*p* < 0.01). The expression levels of target gene in the heart, liver, spleen, lung, and kidney of mice were not significantly different from that of the controls (*p* > 0.05).

### Mastitis Pathogen Verification and Colony-Forming Units

**Figure 6:**
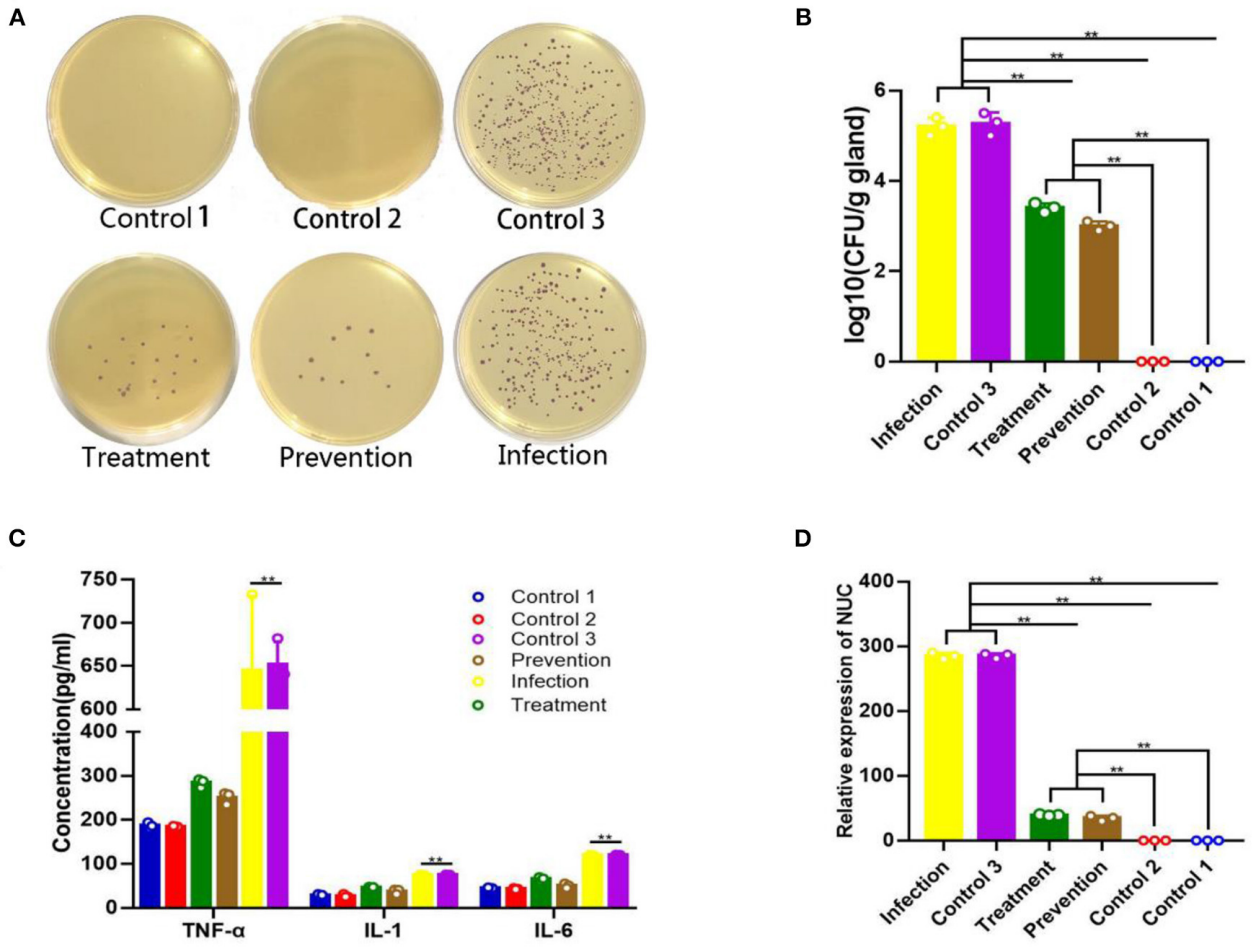
Therapeutic effect of plasmid pBLG-TAP-EGFP-N1 on *S. aureus*-infected mice. **(A)** Chromogenic culture plate colony count of *S. aureus* in murine mammary tissue homogenate. The number of plate colonies in the treatment (*S. aureus*+TAP plasmid, *n* = 3) and prevention (TAP plasmid+*S. aureus, n* = 3) groups were significantly lower than that in the infection group. The number of plate colonies in control groups 2 (Empty vector, *n* = 3) and 3 (*S. aureus*+Empty vector, *n* = 3) showed that the empty vector neither caused nor had any resistance to *S. aureus* infection in the mammary glands of mice. **(B)** Mammary gland CFU post plasmid pBLG-TAP-EGFP-N1 treatment. The CFU of the mammary gland was highly consistent with the above results, which further supported the above conclusion. **(C)** Inflammatory cytokine levels in the murine mammary gland. There was no significant difference in the levels of inflammatory factors between the infection group and the control group 3, and both were significantly higher than the other experimental groups. Inflammatory cytokine levels in the treatment and prophylaxis groups were not significantly different from those in uninfected controls 1 (PBS, *n* = 3) and 2. **(D)** Q-PCR of the Nuc gene in murine mammary gland tissue. The change of relative expression level of Nuc gene was consistent with that of CFU in each experimental group.

At 24 h after treatment, the numbers of *S. aureus* colonies reduced significantly both in the prevention group and treatment group, compared with the infection group (Figure 6A). Mammary glands of mice in the prevention group had a median bacterial load of 3.74 log_10_ CFU/g of gland, and those in the treatment group had a median bacterial load of 3.51 log_10_ CFU/g, which represents an decrease of 5.62 log_10_ CFU/g of gland compared to the original inoculum (Figure 6B). The qPCR showed a reduction of *S. aureus*-specific gene *Nuc* relative expression in the *TAP*-treated group (Figure 6C).

### Histopathological Changes

**Figure 7:**
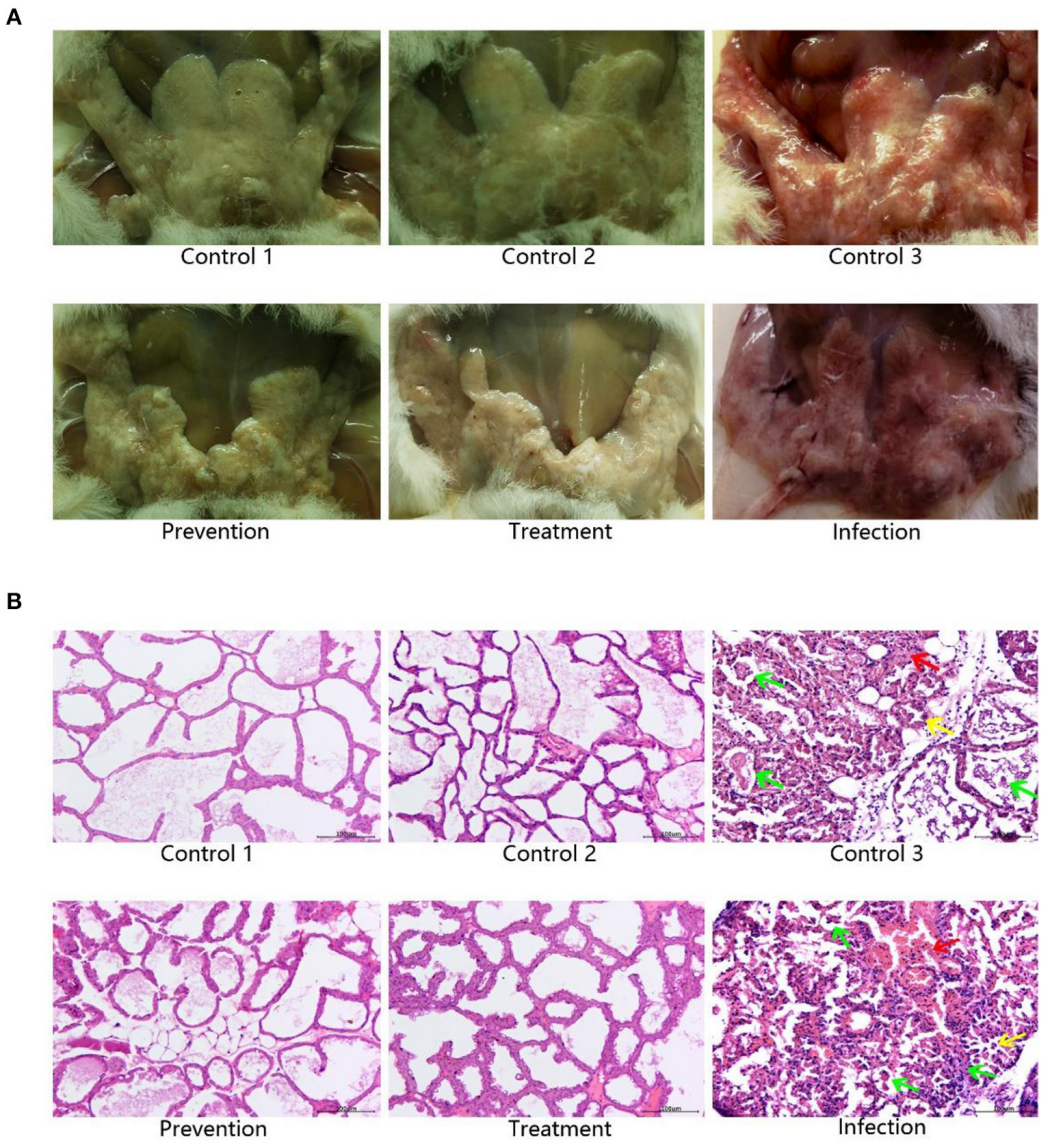
Pathology of mammary glands in mice. **(A)** Gross pathology of murine mammary glands. The murine mammary glands of control 1 (PBS, *n* = 3) and 2 (Empty vector, *n* = 3), which were not infected with *S. aureus*, were milky and lack of inflammation. Mice infected with *S. aureus* in the control 3 (*S. aureus*+Empty vector, *n* = 3) and infection (*S. aureus, n* = 3) groups showed significant red and inflammatory responses in the mammary glands, while those in the treatment (*S. aureus*+*TAP* plasmid, *n* = 3) and prevention (*TAP* plasmid+*S. aureus, n* = 3) groups showed the opposite. **(B)** Histopathological examination of murine mammary glands. Mice infected with *S. aureus* without transfection *TAP* gene showed significant inflammatory symptoms, including inflammatory cell infiltration, local breast acinar hyperplasia, blood vessel dilatation, and bleeding in the control 3 and infection groups. Regardless of whether the *TAP* vector was injected before or after *S. aureus* inoculation, inflammation remission was significant in the treatment and prevention groups. This result was very similar to control 1 and control 2 without *S. aureus* infection. Scale bar: 100 μm. Green arrow: The inflammatory cells were infiltrated. Yellow arrow: Hyperplasia of breast acinus. Red arrow: Vascular dilatation and hemorrhage.

Mammary glands were graded for gross pathological appearance. Mild or lack of inflammation was characterized by the presence of milk in the mammary glands, while redness of the glands indicated a severe local inflammatory reaction accompanied with a slimy exudate, indicative of a high bacterial burden (25). The mammary glands of mice in the model group showed significant and severe inflammation, which were very similar to that in control group 3. The prevention group and treatment group showed mild inflammatory symptoms, which were very similar to those in control groups 1 and 2 (Figure 7A).

We elucidated the therapeutic effect visually through mammary gland paraffin sections. After *S. aureus* infection, inflammation could be easily detected in the sections. The interstitium of mammary gland cells was obviously widened, high level of edema was observed, and a large number of inflammatory cells were infiltrated. The vascular dilatation and hemorrhage were observed. Most of the acinar epithelial cells in the exudated area were necrotic, detached, disintegrated, and disappeared. Neutrophils were scattered in many acinus, and part of the neutrophils were necrotic and disintegrated. Regardless of whether the *TAP* vector was injected before or after *S. aureus* inoculation, inflammation remission was significant. There were a few inflammatory cells in the breast tissue, the infiltration was not obvious, the phenomenon of acinar epithelial abscission and necrosis was less, and the breast cells were basically intact (Figure 7B).

### Changes in Inflammatory Cytokines

Inflammatory cytokines in the breast were measured. As shown in Figure 6D, the levels of TNF-α, IL-1β, and IL-6 after *TAP* plasmid treatment were significantly lower than those of the model group (*p* < 0.001). In particular, the decrease in TNF levels was the most significant, and the levels of IL-1β and IL-6 in the prevention group and treatment group were close to those in control groups 1 and 2. Similarly, the control group 2 did not show a significant increase in inflammatory cytokine levels compared to the control group 1. There was no significant difference between the levels of inflammatory factors in control group 3 and the model group, and the empty vector did not relieve the inflammation of mastitis mice.

## Discussion

Bovine mastitis is a common disease with high incidence, mainly caused by bacterial infection, and *S. aureus* is an important pathogenic bacterium (26). Bovine mastitis results in prevalent and large-scale financial losses in the global dairy industry (4). Small-molecule antibiotic therapy has already been used for both the treatment and prevention of *S. aureus*-induced mastitis. The treatment of bovine mastitis with currently approved antibiotics, such as pirlimycin, has a cure rate of about 10–30% (27). However, there are many drawbacks to using antibiotics to treat mastitis in cows (28). The presence of antibiotic residues in milk intended for human consumption is a matter of consumer concern. Antibiotics in milk were first identified in the 1960s and have been increasing ever since, with a significant increase in detection since 2000 (29). In order to avoid the problem of residual milk after antibiotics are used, farms often choose to stop production for a period of time after antibiotics are given, resulting in milk returning to antibiotic-free levels. Such a measure is obviously not the most satisfactory option for farmers, and it will bring serious economic losses. In addition, the problem of bacterial resistance caused by long-term use of antibiotics has also been a major concern. Global concerns regarding bacterial antibiotic resistance demand prudent use of antibiotics in livestock production (30). The resistance rates of *S. aureus* infection and multidrug-resistant strains are increasing, making the clinical anti-infective treatment more difficult (31). As a result, it is particularly urgent and important to develop new drugs and alternative antibiotic therapies (32).

Antimicrobial peptide (AMP) is an important immunomodulated molecule that can protect the host from the invasion of pathogenic bacteria (33) and has been widely considered as a new alternative antibiotic product. Unfortunately, despite the potent antibacterial activity of AMPs *in vitro*, the hysteretic screening approach and *in vivo* failure still hinder their further development, thus becoming the largest barrier to their clinical application. For example, pig protegrin (34) derivative IB-367 (iseganan) failed in Phase III clinical trials of oral mucositis (35, 36). Similarly, magainin can be easily degraded by various proteases in blood circulation within a few minutes, thereby losing its antimicrobial activity (37). A series of factors, including serum stability, toxicity, and the production cost, is far from negligible when it comes to clinical application and *in vivo* efficacy. In recent years, many gene expression vector delivery strategies have emerged in the treatment of inflammatory diseases. For example, IL-19 gene recombinant plasmid was used to treat experimental autoimmune myocarditis in rats (38). The results showed that the recombinant plasmid could significantly inhibit the inflammatory response of experimental autoimmune myocarditis in rats. Local injection of naked plasmid DNA encoding miR-200c in gingival tissues effectively rescued miR-200c expression, protected against periodontal inflammation, and alleviated the impaired glucose metabolism in PiOS mice (39). This strategy makes up for the regret of giving AMPs directly and provides us with a new idea of using AMPs. However, some existing plasmid delivery strategies still have some disadvantages. The recombinant plasmid cannot be expressed directionally in the lesion, and the expression of exogenous genes in other tissues or organs may affect the health level of the body and lead to other diseases (40–42). In the treatment of cow mastitis, several researchers have performed related application exploration. Chen et al. constructed the recombinant plasmid carrying human-derived lysozyme gene for the treatment of bovine mastitis (43). However, the introduction of exogenous gene will increase the concern of biosafety and may induce some negative effects on the production of animals (44, 45). In contrast, in this study, we constructed a mammary gland tissue-specific expression vector carrying the AMP of bovine-derived *TAP* gene and evaluated its expression efficiency in pBMECs, HEK293T cells, and different organs of mice. In this way, it will also avoid the potential biosafety risk and immunological rejection of the recombinant plasmid being expressed in other tissues. TAP is a member of the β-defensin family of antibiotic peptides, which is expressed in the tracheal mucosa and mammary gland of cows, and had a broad-spectrum antibacterial effect (14). There are a lot of other studies that have been done on β-defensins, demonstrating their therapeutic potential in inflammatory diseases. Equine β-defensin 1 (EBD1) is induced in the respiratory tract in response to equine herpes virus infections (46). Porcine β-defensin 2 affects bacteria and pseudorabies virus (47). P9 peptide derived from mouse β-defensin-4 makes aggregation with viral glycoproteins, which prevent viral RNA release, and P9 has been reported to have a broad range of antiviral properties against various respiratory viruses (48). The rat β-defensin-2 and IL-22 emerged as biomarkers for the multidrug-resistant *Klebsiella pneumoniae* bacterial infection (49).

The overexpression vector often carries *GFP* and other marker genes, and the expression product is a fusion protein with GFP. Therefore, the effect of tagging proteins on the function of AMPs should be considered in advance. The TAP involved in this study is an amphiphilic polypeptide, which exists in the form of polypeptide. This prevents its non-polar surface from being exposed to aqueous solutions. Defensins acts on the membrane or envelope wall by its amphiphilicity (50–52). The classical mechanism of action of cationic defensins is the disturbance of the anionic bacterial membrane. In this way, bacterial destruction occurs by the interaction between the electrostatic forces of positively charged amino acids within defensins(s) and the negatively charged ones on the cellular surface (53). The structure of GFP is very stable. The spatial structure is cylindrical, just like a bucket, and the luminescent group is located in the center of the bucket. Thus, we can visualize it as a paint bucket with pigment. The outer wall of the bucket effectively prevents its influence on the spatial structure of the target protein (54–56). The GFP does not require any reaction substrate and cofactor, has no species restriction, and can be expressed in a variety of biological cells to emit stable fluorescence. Existing experiments have proved that GFP does not affect the bactericidal effect of defensins (57, 58). In conclusion, we can conclude that GFP does not affect the spatial structure and bactericidal mechanism of TAP. The therapeutic effect of TAP in mice increased the possibility of its foreseeable application in the treatment of bovine mastitis.

For the studies *in vitro*, the recombinant plasmid can be specially expressed in the targeted cells, bovine mammary epithelial cells. In addition, a significant protective effect on preventing the apoptosis of mammary epithelial cells infected by *S. aureus* was observed. Although a certain degree of cell necrosis was induced by transfection of the recombinant plasmid, this effect was limited. This may be due to the cytotoxicity of liposomes at the initial stage of transfection. When the AMPs were expressed, they prevented cell apoptosis caused by bacterial infection. *In vivo* studies in mice, quantitative bacterial loads, histological evaluation, the expression levels of inflammatory factors, and the differences of qualitative clinical symptoms all strongly indicated that the treatment with plasmid pBLG-TAP-EGFP-N1 significantly decreased the intramammary bacterial loads and inflammation caused by *S. aureus*-induced bovine mastitis. Moreover, the q-PCR and Western blot indicated that the recombinant plasmid was able to significantly increase the expression level of *TAP* gene in murine mammary gland without spreading systemically. Of course, we detected extremely obscure protein bands in tissues other than the breast, which we can attribute to a guess that the target protein travels through the bloodstream into other tissues in small amounts, so this level of expression is negligible. It is worth mentioning that the empty vector was introduced as the control group, and we found that transfection of the empty vector neither caused the inflammation of the mouse breast, nor had the effect of relieving the breast inflammation induced by *S. aureus*. On the premise that the interfering factors are fully eliminated, we can basically confirm that TAP produces an obvious effect on the treatment of mastitis in mice.

In summary, the ultimate goal of the dairy industry is to produce reliable and high-quality dairy products for customers, which is undeniably profit-oriented. Therefore, low-cost and mature approaches to attenuate the effect of both mastitis and antibiotic overuse are truly feasible ways to address this difficulty. This study demonstrated a proof of principle that plasmid-delivered AMPs could reduce mammary bacterial infection in mice. Further studies are needed to evaluate the biosafety and antibacterial effect of pBLG-TAP-EGFP-N1 in cows with mastitis.

## Data Availability Statement

The raw data supporting the conclusions of this article will be made available by the authors, without undue reservation.

## Ethics Statement

The animal study was reviewed and approved by Experimental Animal Ethics Committee of Yangzhou University.

## Author Contributions

ZZ, YY, and ZY conceived and designed the experiments. ZZ, DC, and XL performed the experiments. ZZ, ML, TX, ZY, and YM analyzed the data. ZZ, ZC, and RZ wrote the original manuscript. ZZ, YY, and ZY revised the manuscript. All authors contributed to the article and approved the submitted version.

## Funding

This research was supported by the Jiangsu Agriculture Science and Technology Innovation Fund [Grant No. CX(20)3089], the National Natural Science Foundation of China [Grant Nos. 31872324 and 32002263], the National Natural Science Foundation of China (Grant No. 32002263), and the Basic Research Program of Jiangsu Province (Grant No. BK20190881).

## Conflict of Interest

The authors declare that the research was conducted in the absence of any commercial or financial relationships that could be construed as a potential conflict of interest.

## Publisher's Note

All claims expressed in this article are solely those of the authors and do not necessarily represent those of their affiliated organizations, or those of the publisher, the editors and the reviewers. Any product that may be evaluated in this article, or claim that may be made by its manufacturer, is not guaranteed or endorsed by the publisher.

## References

[1] Ruegg PL. 100-Year Review: Mastitis detection, management, and prevention. J Dairy Sci. (2017) 100:10381–97. 10.3168/jds.2017-13023 29153171

[2] Molineri A Camussone C Zbrun MV Archilla G Cristiani M Neder V. Antimicrobial resistance of Staphylococcus aureus isolated from bovine mastitis: systematic review and meta-analysis. Prev Vet Med. (2021) 188:105261. 10.1016/j.prevetmed.2021.105261 33508662

[3] Thompson-Crispi K Atalla H Miglior F Mallard BA. Bovine mastitis: frontiers in immunogenetics. Front Immunol. (2014) 5:493. 10.3389/fimmu.2014.00493 25339959 PMC4188034

[4] Breyne K Honaker RW Hobbs Z Richter M Zaczek M Spangler T. Efficacy and Safety of a Bovine-Associated Staphylococcus aureus Phage Cocktail in a Murine Model of Mastitis. Front Microbiol. (2017) 8:2348. 10.3389/fmicb.2017.02348 29234314 PMC5712351

[5] Reshi AA Husain I Bhat SA Rehman MU Razak R Bilal S. Bovine mastitis as an evolving disease and its impact on the dairy industry. Int J Cur Res Rev. (2015) 7:48–55. 10.4028/www.scientific.net/SSP.134.57

[6] Curone G Filipe J Cremonesi P Trevisi E Amadori M Pollera C. What we have lost: mastitis resistance in Holstein Friesians and in a local cattle breed. Res Vet Sci. (2018) 116:88–98. 10.1016/j.rvsc.2017.11.020 29223308

[7] Berghash SR Davidson JN Armstrong JC Dunny GM. Effects of antibiotic treatment of nonlactating dairy cows on antibiotic resistance patterns of bovine mastitis pathogens. Antimicrobial Agents Chemother. (1983) 24:771–6. 10.1128/AAC.24.5.771 6660851 PMC185940

[8] Jian G Fu-Qing Y Li-Ping L Jian-Zhong H Rong-Guang H Han-Qi Z. Antibiotic resistance of Streptococcus agalactiae from cows with mastitis. Veterinary J. (2012) 194:423–4. 10.1016/j.tvjl.2012.04.020 22627045

[9] Gutiérrez D Garrido V Fernández L Portilla S Rodriguez A Grillo MJ. Phage lytic protein LysRODI prevents staphylococcal mastitis in mice. Front Microbiol. (2020) 11:7. 10.3389/fmicb.2020.00007 32038593 PMC6989612

[10] Dehkordi SH Hosseinpour F Kahrizangi AE. An *in vitro* evaluation of antibacterial effect of silver nanoparticles on Staphylococcus aureus isolated from bovine subclinical mastitis. Afr J Biotechnol. (2011) 10:10795. 10.5897/AJB11.1499

[11] Alluwaimi AM. The cytokines of bovine mammary gland: prospects for diagnosis and therapy. Res Vet Sci. (2004) 77:211–22. 10.1016/j.rvsc.2004.04.006 15276772

[12] Domadia P Swarup S Bhunia A Sivaraman J Dasgupta D. Inhibition of bacterial cell division protein FtsZ by cinnamaldehyde. Biochem Pharmacol. (2007) 74:831–40. 10.1016/j.bcp.2007.06.029 17662960

[13] Laport MS Marinho PR Santos OCDS Almeida PD Romanos MTV Muricy G. Antimicrobial activity of marine sponges against coagulase-negative staphylococci isolated from bovine mastitis. Vet Microbiol. (2012) 155:362–8. 10.1016/j.vetmic.2011.09.004 21958748

[14] Taha-Abdelaziz K Perez-Casal J Schott C Hsiao J Attah-Poku S Slavic D. Bactericidal activity of tracheal antimicrobial peptide against respiratory pathogens of cattle. Vet Immunol Immunopathol. (2013) 152:289–94. 10.1016/j.vetimm.2012.12.016 23333196

[15] Aono S Li C Zhang G Kemppainen RJ Gard J Lu W. Molecular and functional characterization of bovine beta-defensins-1. Vet Immunol Immunopathol. (2006) 113:181–90. 10.1016/j.vetimm.2006.05.002 16777238

[16] Diamond G Zasloff M Eck H Brasseur M Maloy WL Bevins CL. Tracheal antimicrobial peptide, a cysteine-rich peptide from mammalian tracheal mucosa: Peptide isolation and cloning of a cDNA. Proc Natl Acad Sci USA. (1991) 88:3952–6. 10.1073/pnas.88.9.3952 2023943 PMC51571

[17] López-Meza JE Gutiérrez-Barroso A Ochoa-Zarzosa A. Expression of tracheal antimicrobial peptide in bovine mammary epithelial cells. Res Vet Sci. (2009) 87:59–63. 10.1016/j.rvsc.2008.12.005 19181355

[18] Chen Z Chu S Wang X Sun Y Xu T Mao Y. MiR-16a regulates milk fat metabolism by targeting large tumor suppressor kinase 1 (LATS1) in bovine mammary epithelial cells. J Agric Food Chem. (2019) 67:11167–78. 10.1021/acs.jafc.9b04883 31542928

[19] Canovas J Baldry M Bojer MS Andersen PS Gless BH Grzeskowiak PK. Cross-talk between staphylococcus aureus and other staphylococcal species via the agr quorum sensing system. Front Microbiol. (2016) 7:1733. 10.3389/fmicb.2016.01733 27877157 PMC5099252

[20] Li WH Li Y Chu Y Wu WM Yu QH Zhu XB. PLCE1 promotes myocardial ischemia-reperfusion injury in H/R H9c2 cells and I/R rats by promoting inflammation. Biosci Rep. (2019) 39:7. 10.1042/BSR20181613 31217261 PMC6609553

[21] Yao H Zhang Y He F Wang C Xiao Z Zou J. Short hairpin RNA targeting 2B gene of coxsackievirus B3 exhibits potential antiviral effects both *in vitro* and *in vivo*. BMC Infect Dis. (2012) 12:177. 10.1186/1471-2334-12-177 22863145 PMC3482581

[22] Pereyra EAL Sacco SC Dure A Baravalle C Renna MS Andreotti CS. Immune response of Staphylococcus aureus strains in a mouse mastitis model is linked to adaptive capacity and genotypic profiles. Vet Microbiol. (2017) 204:64–76. 10.1016/j.vetmic.2017.04.009 28532808

[23] Han Z Fan Y Yang Z Loor JJ Yang Y. Mammary transcriptome profile during peak and late lactation reveals differentially expression genes related to inflammation and immunity in chinese holstein. Animals. (2020) 10:510–24. 10.3390/ani10030510 32204353 PMC7143190

[24] Skowron K Wałecka-Zacharska E Grudlewska K Kwiecinska-Pirog J Wiktorczyk N Kowalska M. Effect of selected environmental factors on the microbicidal effectiveness of radiant catalytic ionization. Front Microbiol. (2020) 10:3057. 10.3389/fmicb.2019.03057 32038531 PMC6989485

[25] Notebaert S Demon D Berghe TV Vandenabeele P Meyer E. Inflammatory mediators in Escherichia coli-induced mastitis in mice. Comp Immunol Microbiol Infect Dis. (2008) 31:551–65. 10.1016/j.cimid.2007.10.004 18243314

[26] Heimes A Brodhagen J Weikard R Seyfert HM Becker D Meyerholz MM. Hepatic transcriptome analysis identifies divergent pathogen-specific targeting-strategies to modulate the innate immune system in response to intramammary infection. Front Immunol. (2020) 11:715. 10.3389/fimmu.2020.00715 32411137 PMC7202451

[27] Gomes F Henriques M. Control of bovine mastitis: old and recent therapeutic approaches. Curr Microbiol. (2016) 72:377–82. 10.1007/s00284-015-0958-8 26687332

[28] Manyi-Loh C Mamphweli S Meyer E Okoh A. Antibiotic use in agriculture and its consequential resistance in environmental sources: potential public health implications. Molecules. (2018) 23:795. 10.3390/molecules23040795 29601469 PMC6017557

[29] Lu G Chen Q Li Y Liu Y Zhang Y Hhuang Y. Status of antibiotic residues and detection techniques used in Chinese milk: A systematic review based on cross-sectional surveillance data. Food Research International. (2021) 147:0963–9969. 10.1016/j.foodres.2021.110450 34399452

[30] Rajala-Schultz P Nødtvedt A Halasa T Waller KP. Prudent use of antibiotics in dairy cows: the nordic approach to udder health. Front Veterinary Sci. (2021) 8:623998. 10.3389/fvets.2021.623998 33748209 PMC7973009

[31] Guo Y Song G Sun M Wang J Wang Y. Prevalence and Therapies of Antibiotic-Resistance in Staphylococcus aureus. Front Cell Infect Microbiol. (2020) 10:107. 10.3389/fcimb.2020.00107 32257966 PMC7089872

[32] Founou LL Founou RC Essack SY. Antibiotic resistance in the food chain: a developing country-perspective. Front Microbiol. (2016) 7:1881. 10.3389/fmicb.2016.01881 27933044 PMC5120092

[33] Liu Y Shi J Tong Z Jia Y Yang B Wang Z. The revitalization of antimicrobial peptides in the resistance era. Pharmacol Res. (2021) 163:1043–6618. 10.1016/j.phrs.2020.105276 33161137

[34] Bellm L Lehrer RI Ganz T. Protegrins: new antibiotics of mammalian origin. Expert Opin Investig Drugs. (2000) 9:1731–42. 10.1517/13543784.9.8.1731 11060772

[35] Giles FJ Miller CB Hurd DD Wingard JR Fleming TR Sonis ST. A phase III, randomized, double-blind, placebo-controlled, multinational trial of iseganan for the prevention of oral mucositis in patients receiving stomatotoxic chemotherapy (PROMPT-CT trial). Leuk Lymphoma. (2003) 44:1165–72. 10.1080/1042819031000079159 12916869

[36] Trotti A Garden A Warde P Symonds P Langer C Redman R. A multinational, randomized phase III trial of iseganan HCl oral solution for reducing the severity of oral mucositis in patients receiving radiotherapy for head-andneck malignancy. Int J Radiat Biol. (2004) 58:674–81. 10.1016/S0360-3016(03)01627-4 14967419

[37] Meng H Kumar K. Antimicrobial activity and protease stability of peptides containing fluorinated amino acids. J Am Chem Soc. (2007) 129:15615–22. 10.1021/ja075373f 18041836

[38] Chang H Zhao F Wang Y Li G Zhang L Zou J. Effects of intravenous injection of IL-19 recombinant plasmid on the treatment of experimental autoimmune myocarditis in rats. Chin J Pathophysiol. (2015) 31:744–9.

[39] Krongbaramee T Zhu M Qian Q Zhang Z Eliason S Shu Y. Plasmid encoding microRNA-200c ameliorates periodontitis and systemic inflammation in obese mice. Molec Therapy Nucleic Acids. (2021) 23:1204–16. 10.1016/j.omtn.2021.01.030 33664998 PMC7899952

[40] Ismail R Allaudin ZN Lila MAM. Scaling-up recombinant plasmid DNA for clinical trial: Current concern, solution and status. Vaccine. (2012) 30:5914–20. 10.1016/j.vaccine.2012.02.061 22406276

[41] Williams JA Carnes AE Hodgson CP. Plasmid DNA vaccine vector design: impact on efficacy, safety and upstream production. Biotechnol Adv. (2009) 27:353–70. 10.1016/j.biotechadv.2009.02.003 19233255 PMC2693335

[42] Smith HA Klinman DM. The regulation of DNA vaccines. Curr Opin Biotechnol. (2001) 12:299–303. 10.1016/S0958-1669(00)00215-9 11404109

[43] Shen C Lin Y Ye CR Jin YZ Yu XQ Sun HC. Treatment of milk cow mastitis with recombinant lysozyme plasmid pcdNAKLYZ. Chin Dairy Cow. (2011) 04:4–6.

[44] Manders P Thomas R. Immunology of DNA vaccines: CpG motifs and antigen presentation. Inflamm Res. (2000) 49:199–205. 10.1007/s000110050580 10893042

[45] Scheule RK. The role of CpG motifs in immunostimulation and gene therapy. Adv Drug Deliv Rev. (2000) 44:119–34. 10.1016/S0169-409X(00)00090-9 11072110

[46] Cleemput J V Poelaert K C Laval K. Alphaher pesvirus exploits antimicrobial β-1 defensinss to initiate respiratory tract infection. J Virol. (2020) 94:01676–19. 10.1128/JVI.01676-19 31996426 PMC7108845

[47] Huang J Qi Y Wang A Huang C Liu X Yang X. Porcine β-defensins 2 inhibits proliferation of pseudorabies virus *in vitro* and in transgenic mice. Virol J. (2020) 17:1–7. 10.1186/s12985-020-1288-4 32014007 PMC6998849

[48] Zhao H Zhou J Zhang K Chu H Liu D Poon VKM. A novel peptide with potent and broad-spectrum antiviral activities against multiple respiratory viruses. Sci Rep. (2016) 6:22008. 10.1038/srep22008 26911565 PMC4766503

[49] Fan J Luo Y Qin Y Wu C Han X Quyang H. The expression of β-Defensins-2, IL-22, IL-22R1 and IL-10R2 in rat model of Klebsiella pneumonia and their correlation with histological grades. Exp Lung Res. (2020) 46:109–16. 10.1080/01902148.2020.1725690 32169023

[50] Singh PK Jia HP Wiles K Hesselberth J Liu L Conway BAD. Production of β-defensinss by human airway epithelia. Proc Natl Acad Sci Unit States Am. (1998) 95:14961–6. 10.1073/pnas.95.25.14961 9843998 PMC24558

[51] Semple F Dorin JR. β-Defensinss: multifunctional modulators of infection. inflammation and more? J Innate Immun. (2012) 4:337–48. 10.1159/000336619 22441423 PMC6784047

[52] Prasad SV Fiedoruk K Daniluk T Piktel E Bucki R. Expression and function of host defense peptides at inflammation sites. Int J Mol Sci. (2020) 21:104. 10.3390/ijms21010104 31877866 PMC6982121

[53] Solanki SS Singh P Kashyap P Sansi MS Ali SA. Promising role of defensinss peptides as therapeutics to combat against viral infection. Microb Pathog. (2021) 155:0882–4010. 10.1016/j.micpath.2021.104930 33933603 PMC8084285

[54] Olenginski GM Piacentini J Harris DR Runko NA Papoutsis BM Alter JR. Structural and spectrophotometric investigation of two unnatural amino-acid altered chromophores in the superfolder green fluorescent protein. Acta Crystallogr D Struct Biol. (2021) 77:1010–8. 10.1107/S2059798321006525 34342274 PMC8329867

[55] Chen C Tachibana SR Baleeva NS Myasnyanko IN Bogdanov AM Gavrikov AS. Developing bright green fluorescent protein (GFP) -like fluorogens for live-cell imaging with nonpolar protein-chromophore interactions. Chemistry-A Eur J. (2021) 27:8946–50. 10.1002/chem.202101250 33938061

[56] Kong J Wang Y Qi W Huang M Su R He Z. Green fluorescent protein inspired fluorophores. Adv Colloid Interface Sci. (2020) 285:102286–7. 10.1016/j.cis.2020.102286 33164780

[57] Geng F Wang A Chen F Shen M Chen M Su Y. Establishment of a human papillomavirus pseudovirus in vitro infection model and its application to evaluate antiviral activity of human defensins-5. Acta Academiae Med Militaris Tertiae. (2012) 34:473–6. 10.16016/j.1000-5404.2012.06.002

[58] Sousa A Almeida AM Faria R Konate K Boisguerin P Queiroz JA. Optimization of peptide-plasmid DNA vectors formulation for gene delivery in cancer therapy exploring design of experiments. Colloids Surf B: Biointerfaces. (2019) 183:110417–8. 10.1016/j.colsurfb.2019.110417 31408780

